# Are more experienced clinicians better able to tolerate uncertainty and manage risks? A vignette study of doctors in three NHS emergency departments in England

**DOI:** 10.1136/bmjqs-2018-008390

**Published:** 2019-02-06

**Authors:** Rebecca Lawton, Olivia Robinson, Rebecca Harrison, Suzanne Mason, Mark Conner, Brad Wilson

**Affiliations:** 1 Institute of Psychological Sciences, University of Leeds, Leeds, UK; 2 Quality and Safety Research, Bradford Institute for Health Research, Bradford, UK; 3 School of Psychology, University of Leeds, Leeds, UK; 4 School of Health and Related Research, University of Sheffield, Sheffield, UK; 5 Accident and Emergency, Bradford Teaching Hospitals NHS Foundation Trust, Bradford, UK

**Keywords:** decision making, emergency department, cognitive biases, patient safety

## Abstract

**Background:**

Risk aversion among junior doctors that manifests as greater intervention (ordering of tests, diagnostic procedures and so on) has been proposed as one of the possible causes for increased pressure in emergency departments (EDs). Here we tested the prediction that doctors with more experience would be more tolerant of uncertainty and therefore less risk-averse in decision making.

**Methods:**

In this cross-sectional, vignette-based study, doctors working in three EDs were asked to complete a questionnaire measuring experience (length of service in EDs), reactions to uncertainty (Gerrity *et al*, 1995) and risk aversion (responses about the appropriateness of patient management decisions).

**Results:**

Data from 90 doctors were analysed. Doctors had worked in the ED for between 5 weeks and 21 years. We found a large association between experience and risk aversion so that more experienced clinicians made less risk-averse decisions (r=0.47, p<0.001). We also found a large association between experience and reactions to uncertainty (r=−0.50, p<0.001), with more experienced doctors being much more at ease with uncertainty. Mediation analyses indicated that tolerance of uncertainty partially mediated the relationship between experience and lower risk aversion, explaining about a quarter of the effect.

**Conclusion:**

While we might be tempted to conclude from this research that experience and the ability to tolerate uncertainty lead to positive outcomes for patients (less risk-averse management strategies and higher levels of safety netting), what we are unable to conclude from this design is that these less risk-averse strategies improve patient safety.

## Introduction

Few can deny that emergency departments (EDs) in England are currently managing unprecedented levels of demand with over 40 000 people attending a major, or type 1, ED each day across the National Health Service (NHS) in 2015–2016.^1^ This increase in demand has resulted in a longer wait for initial assessment and time to the start of treatment.^2^ In order to reduce patient waiting times, the Department of Health reformed emergency care, emphasising nobody should wait more than 4 hours in the ED from arrival to admission, transfer or discharge. While evidence suggests this has led to a reduced waiting time by 30%, some have argued that the implementation of the 4-hour target has encouraged individuals with minor conditions to visit EDs more frequently as they are seen quicker than booking appointments with a local general practitioner (GP).^3^ Gibney *et al* 4 argue that many EDs manage patients ‘who have not been involved in accidents nor are they emergencies’. Despite these concerns, and the likelihood that both factors have contributed to increased demand on the service, recent evidence suggests that the majority of attendances at ED (80%) are unavoidable.^5^

In this challenging environment, doctors are expected to use their judgement to deliver high-quality and safe care while at the same time managing patient throughput and waiting times.

Clinical decision making has been defined as the judgements made on the presence, type, severity and treatment of patient illnesses.^6^ In some cases medical professionals may be forced to make clinical decisions despite not having all the information at hand or being uncertain of the best course of action. Modern theories of decision making have moved beyond the notion that reasoning is entirely logical and rational (‘analytical’ or system 2 thinking) and acknowledge the important influence of feelings in the decisions we make. This ‘experiential or system 1’ relies on previous experiences, images and associations that are bound up in emotion and affect so that a decision becomes more a feeling of whether something is good or bad.^7^ One might predict then that more experienced clinicians process situations quickly and rely on ‘vibes’ based on their previous experience and intuition. On the other hand, less experienced clinicians, according to this theory, will be more deliberative, analytical and will favour delayed or slower action.

In a clinical setting, Juliusson *et al* 8 demonstrated that past experiences can impact future decision making. In particular, those with additional years of experience are able to develop the ability to intuitively know what to do and to quickly recognise critical aspects of a situation.^9^ Hausmann and colleagues^10^ found when tracing the diagnostic process of physicians in an ED that the more experienced physicians needed fewer contextual cues to verify a suspected diagnosis and that ‘case experience’ had an effect on the confidence rating of the final diagnosis. In other words, doctors use their experience in order to identify a reasonable course of action, spending less time weighing alternative decisions^11^ and using fewer resources when making diagnostic decisions. The naturalistic decision-making approach^12^ also asserts that doctors and nurses use past experiences and ‘pattern matching’ in order to make emergency decisions, to rapidly generate option strategies for patients,^6^ and that this in turn can lead to an increase in patient turnover and less use of NHS resources. Furthermore, a recent NHS report^2^ identifies risk aversion among junior doctors that manifests as greater intervention (ordering of tests, diagnostic procedures and so on) as one of the possible causes for increased pressure in EDs. Thus, hypothesis 1, tested in this study, is that *more years of experience in the ED will be associated with less risk-averse decisions*.

As well as experience, another variable purported to be related to decision-making style is the individual characteristic ‘tolerance of uncertainty’. A study by Tsiga and Panagopoulou^13^ found GPs respond to uncertainty by increasing hospital referrals and ordering more diagnostic tests. In addition to this, GPs that are more intolerant of uncertainty also have a higher cost of investigation and treatment.^14^ Research by Bachman and Freeborn^15^ acknowledged that physicians who were uncertain about making a clinical decision on behalf of a patient demonstrated increased referrals. In addition to this, Allison and colleagues^16^ found an uncertainty–response relationship in primary care physicians in the USA, with increased physician anxiety being associated with higher charges for the patient and higher resource use. In another study family physicians, who were identified to be less risk-averse than hospital internists, generated 5% lower costs.^17^ This evidence suggests that those doctors who demonstrate a lower tolerance of uncertainty will be more likely to adopt slower, more deliberative decision making which may be associated with management decisions that are risk-averse, for example, ordering further tests or admitting patients for observation. Thus, hypothesis 2 is *that lower tolerance of uncertainty will be associated with more risk-averse decision making (involving further tests and* *procedures)*.

Assuming these associations did exist, we further tested whether tolerance of uncertainty explained the relationship between experience and risk aversion in clinical decision making (hypothesis 3). In other words, were doctors with more experience more tolerant of uncertainty and therefore less risk‐averse in decision making?

## Methods

### Participants

One hundred and twenty physicians of all grades were approached to take part in this study from across three EDs in large cities in the North of England, UK (to detect a small effect [0.15], power of 0.95 and p<0.05, with two predictor variables, 74 participants were required). The clinical lead in each department introduced the researcher, who then approached physicians in staff areas during periods of free time over several days in each department. Participants were given a participant information sheet and consent form. Once they had read the sheet and agreed to take part, they were asked to complete a questionnaire about clinical decision making in the ED and to return it either directly to the researcher or to use a return box in the staff room. The questionnaire was designed to minimise the burden on staff. It took approximately 10 min to complete.

### Study design

This was a cross-sectional, vignette-based study. Participants were asked to read four vignettes describing a clinical presentation, and they were then provided with four different management plans. All four vignettes and management plans were developed by the clinical author (BW) and verified by SM to ensure validity across settings (see online supplementary appendix 1 for full details of vignettes 2–4). They were developed so that the ‘correct’ management plan was somewhat ambiguous and a number of options might be deemed clinically acceptable. Two of the management plans in each case were risk-averse, requiring further tests and/or admission of the patient to hospital, two were less risk-averse, usually involving referring a patient back to the GP or offering reassurance and safety netting advice. For example, vignette 1:

10.1136/bmjqs-2018-008390.supp1 Supplementary data

A 27-year-old man presents with fever, sore throat and headache. Observations at triage show a pulse of 109 beats per minute, blood pressure of 127/88, Respiratory Rate (RR) of 16, oxygen saturation (SpO_2_) of 98% and a temperature of 38.4°C. The patient has no significant previous medical history (PMH) and denies regular medication except paracetamol. Examination reveals a well-nourished patient with no coryzal features. He has no neck stiffness or photophobia. There is erythema in the throat and mild exudate on both tonsils. His chest is clear to auscultation and abdomen soft and non-tender.

The four management options in this case were:

- Redirect to own GP;
- Reassure, educate about viral illness, manage with hydration, rest and paracetamol, and follow-up with own GP if not improving or worsening after 7–10 days;
- Perform monospot and full blood count (FBC), manage appropriately with results including antibiotics for tonsillitis, hydration and paracetamol, and follow-up with own doctor in 7–10 days if worsening or not improving;
- Start sepsis pathway and plan for admission.

Participants were asked to rate the extent to which they agreed with each of the management plans on a scale from 1 to 5 (1=strongly disagree to 5=strongly agree). Responses to the management plans that represented high risk aversion (3 and 4 above) were recoded, so that a higher score represented greater risk taking. As the focus was on overall risk taking, we created a single score across the four ratings for the four scenarios (Cronbach’s alpha=0.77 for 16 items) to create an average risk taking score for each physician.

After providing responses to the four clinical vignettes, participants were asked to complete a 15-item ‘Physicians Reactions to Uncertainty’ scale, developed and validated by Gerrity *et al*,^18 19^ which measures four components of tolerance to uncertainty: anxiety about uncertainty, concerns about bad outcomes, reluctance to disclose uncertainty to patients and reluctance to disclose mistakes to other physicians. Unlike other scales in this field, it was developed specifically for studies in a clinical environment. The scale measures emotional reactions and concerns, as well as coping behaviours. Participants are asked to rate the extent to which they agree with each of the 15 items (eg, I usually feel anxious when I am not sure of a diagnosis) on a 6-point scale from 1=strongly disagree to 6=strongly agree. Four items (eg, I am quite comfortable with the uncertainty of patient care) were recoded so that a higher score always indicated a lower tolerance of uncertainty. The overall scale was reliable (Cronbach’s alpha=0.91 for 15 items). A Physician reactions to uncertainty score was created by averaging across all available ratings, with higher scores indicating lower tolerance of uncertainty. Finally participants were asked to indicate the number of years they had worked within an ED setting.

### Analysis

Data were analysed in IBM SPSS V.22.

We first examined the mean and SD of experience, reactions to uncertainty and risk aversion measures and their intercorrelation.

We then used the PROCESS macro developed by Hayes^20^ for SPSS to test whether reactions to uncertainty mediated any relationship between experience and risk aversion. This procedure follows the traditional mediation procedures of Baron and Kenny^21^ and additionally tests the strength of the mediated path using a bootstrap method. Such mediation analysis allowed us to test the hypothesised causal chain where one variable (in this case, experience, X) affects a second variable (M: tolerance of uncertainty), which in turn affects a third variable (risk aversion of patient management plans, Y). The intervening variable, M, is the mediator. It ‘mediates’ or explains the relationship between the predictor (X) and the outcome (Y). Mediation analysis involves a series of regressions to test each of the hypothesised relationships between variables (predicting Y from X; predicting M from X; predicting Y from X and M). Further analyses tested whether controlling for the mediator reduces the impact of the predictor on the outcome and the strength of the mediated path. In the PROCESS macro the strength of the mediated path is estimated using a bootstrapping procedure that does not require the distributions to be normal. The mediated path is considered significant if the 95% CI around the estimate does not include 0.

## Results

Ninety-six doctors completed the questionnaire, representing a response rate of 80%. The data for four respondents were not analysed because less than 50% of the questions were complete. Of the 92 remaining responses, 2 had missing data on experience (years), meaning that all analyses had 90 data points. This sample gave us sufficient power to detect small relationships between variables.

Of the sample, 51 were junior doctors, 18 were middle-grade doctors and 22 were consultants (1 did not provide this information). Participants were split—32, 31 and 29—across the three hospitals. The average number of years that doctors in this sample had been working in ED was 5, with a range from just a few weeks to 21 years. Grade of doctor and years of experience were very strongly correlated (r=0.87, p<0.001). Therefore to avoid multicollinearity within the regression analyses and because experience (in years) was measured on an interval scale, we included this variable, rather than grade, in all analyses. Table 1 shows the mean, SD and intercorrelation (using Pearson’s) for the three variables employed in the regression analyses.

**Table 1:** Mean, SD and correlations for all variables

	Experience (years)	Tolerance of uncertainty	Total risk aversion score	Mean	SD
Experience (years)	–	−0.50***	0.47***	5.04	6.11
Tolerance of uncertainty		–	−0.40***	2.83	0.85
Total risk aversion score			–	2.89	0.56

*P<0.05, **p<0.01, ***p<0.001.

The correlation between experience and tolerance of uncertainty was large (r=−0.50). Based on Cohen’s criteria,^22^ the effect size of the relationship between tolerance of uncertainty and risk aversion of management plan was in the medium range (r=−0.40), while the correlation between experience and risk aversion was in the medium-large range (r=0.47).

Regression analysis indicated that experience was a strong predictor of risk aversion (B=0.042, SE=0.0086, p<0.001, 95% CI 0.025 to 0.059), explaining 21.4% of the variance in risk aversion. Greater experience was associated with being less risk-averse. Experience was also a strong predictor of tolerance of uncertainty (B=−0.070, SE=0.013, p<0.001, 95% CI −0.095 to −0.045), explaining 26.4% of the variance in tolerance of uncertainty. This suggests that as one gains experience in a clinical setting, one becomes more tolerant of uncertainty (or perhaps that those who are more tolerant of uncertainty remain longer within the emergency medicine specialty).

**Figure 1:**
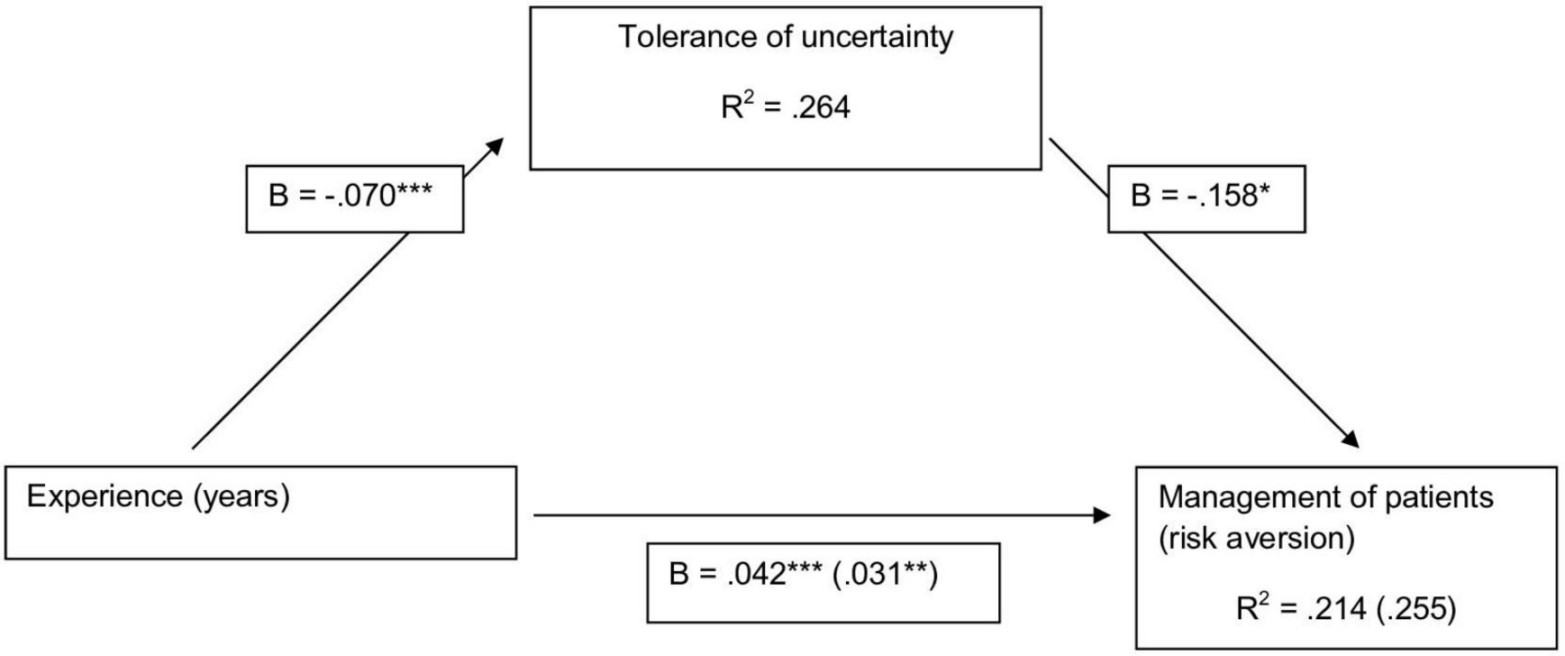
Mediation analysis. All values indicated are unstandardised coefficients. *P<0.05, **p<0.01, ***p<0.001.

Regression analysis also showed that both tolerance of uncertainty (B=−0.158, SE=0.072, p<0.05, 95% CI −0.301 to −0.015) and experience (B=0.031, SE=0.0098, p<0.01, 95% CI 0.012 to 0.051) were significant predictors of risk aversion when entered together (explaining 25.5% of the variance in risk aversion). This supports the idea that tolerance of uncertainty was a partial mediator of the relationship between experience and risk aversion. The mediation analyses are represented as a PATH diagram in figure 1. Based on 1000 resamples the bias-corrected bootstrap estimate of the indirect effect was estimated (B=0.011, SE=0.0063, 95% CI 0.0005 to 0.025). This can be interpreted as a significant mediation effect as the CI did not include 0. Compared with the total effect of experience on risk aversion (B=0.042), approximately a quarter of this effect (B=0.011) was mediated through tolerance of uncertainty and approximately three-quarters of this effect (B=0.031) was not explained by this mediator. The normal theory test of the mediated effect or Sobel test was also significant (B=0.011, SE=0.0055, z=2.02, p<0.05). This tests the reduction in the effect of experience on risk aversion produced by controlling for tolerance of uncertainty. The finding further supports the idea that tolerance of uncertainty is a significant mediator of the relationship between experience and risk aversion.

In other words, the way a clinician chooses to manage a patient can be explained to some extent by how tolerant they are of uncertainty. It can also be explained by the amount of experience they have in ED. However, although as they grow in experience clinicians do become more tolerant of uncertainty, it is not only this tolerance that affects the way they manage patients. There is something else about having more experience that encourages less risk-averse patient management plans.

## Discussion

Eighteen months ago in an article in *T* *he* *New England Journal of Medicine*, Simpkin and Schwartzstein^23^ argued for a new revolution in medicine in which uncertainty is tolerated. They make the point that the consequences for patients when physicians struggle to accept uncertainty are unnecessary tests and the withholding of information. On the other hand, a discomfort with uncertainty can lead to the early closing down of differential diagnoses, meaning that unconscious biases can influence decision making and increase the likelihood of diagnostic error. In this study, we first set out to explore whether those clinicians with more experience in the ED were less risk-averse in their management plans. Second, we investigated whether tolerance of uncertainty might be higher among more experienced clinicians. Third and finally, we tested whether the effect of experience on the preferred approach to the management of patients was mediated (explained) by tolerance of uncertainty.

The first hypothesis we tested in this study was that more experienced doctors would demonstrate a preference for less risk-averse management plans. In other words, more experienced doctors would report less agreement with strategies that involved ordering more tests, offering more treatments and referring more frequently to inpatient services. Our data provided strong support for this hypothesis.

In our sample of 90 doctors in the ED, we found a strong relationship between years of experience and tolerance of uncertainty. There are two possible explanations for this finding. First, it may be the case that those doctors who thrive in EDs and choose to stay in this specialty in the longer term are those that are able to tolerate uncertainty. In other words, people self-select their career specialty based on this personal characteristic. This explanation is a better fit with notions that personality characteristics are relatively stable, enduring traits that are unlikely to be modified over time. The second explanation is that as doctors work for longer in environments where uncertainty is rife, they learn strategies to cope with these feelings. This second explanation is supported by previous research that found that more experienced GPs were more likely to discuss errors with patients than were less experienced staff.^24^ Whichever explanation prevails (and it may be some combination of the two), the fact that those doctors who have spent less time in an ED experience stronger emotional reactions to uncertainty and may feel less willing to disclose this uncertainty or admit to mistakes has important implications for service delivery. Less experienced staff are more likely to take longer to reach decisions because they feel more uncomfortable with uncertainty. Anxiety is known to drive more risk-averse decision making as people who are anxious or fearful make more pessimistic judgements about future events.^25^ Indeed, it is not surprising then that less experienced doctors, who might be both more uncertain and more anxious about that uncertainty, may react by attempting to reduce this uncertainty by ordering more tests and/or choosing to admit patients.

However, our findings show that tolerance of uncertainty only partially mediates the effect of experience on the management strategies of doctors. More experienced doctors, irrespective of their ability to tolerate uncertainty, were more likely to adopt strategies that allowed patients to be dealt with more quickly. The most likely explanation for this, based on theories of decision making (see the Introduction section), is that their experience of similar patients and patterns allows them to reach a best guess diagnosis more quickly. In addition to this, they may then be better able to deal emotionally with, and communicate to patients and colleagues, their lack of complete certainty. Of course, this does not rule out the possibility that they may be overly confident about the accuracy of the diagnosis they have made.^26^

Some authors have proposed that we need a different approach to uncertainty among the medical profession and that this begins in undergraduate curricula, with experienced educators modelling ‘for our students the practice of medicine in which it is all right to be uncertain’.^23^ One approach might be for more experienced doctors to model more effectively how to deal with feelings of uncertainty and the strategies they use (eg, safety netting) to do this. Another implication of this research might be the use of senior doctor review (SDR) as a strategy to reduce demand in ED. SDR means that more experienced doctors do the first-line assessment of patients, making decisions about who requires further investigation before handing these tasks over to more junior staff. While there is some evidence to support SDR, with a recent systematic review^27^ concluding that most studies demonstrated ‘improvements in ED performance measures favouring SDR’, there are also associated costs. The training benefits of a rotation through ED, with respect to increases in confidence and feelings of competence,^28^ compared with placements in other specialties, may be undermined by the deployment of SDR.

Moreover, while we might be tempted to conclude from this research that experience and the ability to tolerate uncertainty lead to positive outcomes for patients (less risk-averse management strategies and higher levels of safety netting), what we are unable to conclude from this design is that these less risk-averse strategies improve diagnostic accuracy and patient safety. This research is limited in two ways: (1) the decisions being assessed are based on hypothetical, although realistic, scenarios, not real-world diagnoses; and (2) we did not measure patient outcomes. Thus, it may be the case that while fewer healthcare resources are used by more experienced doctors and those more tolerant of uncertainty, patients of these doctors are exposed to greater risk. However, Pearson *et al* 29 found no evidence for this in their study of doctors’ decisions to admit patients with acute chest pain. Patients of risk-seeking doctors were just as likely to be alive following discharge as those of risk-averse doctors. The next challenge for applied health research is to explore these relationships in EDs by getting feedback from patients and healthcare records on what happened next. For those working in behavioural medicine or the social sciences, recent conceptual models of tolerance of uncertainty^30^ offer a position from which to better understand how we react to uncertainty. These models will also inform strategies to help both healthcare professionals, whatever their level of experience, and patients cope with the negative thoughts, feelings and responses associated with uncertainty.
