# Supplementary material for: Are more experienced clinicians better able to tolerate uncertainty and manage risks? A vignette study of doctors in three NHS emergency departments in England
Source: BMJ Qual Saf. 2019 Feb 6;28(5):382–8. doi: 10.1136/bmjqs-2018-008390 (PMC6560462; doi:10.1136/bmjqs-2018-008390)
Supplement: Supplementary data [file bmjqs-2018-008390supp001.pdf]

Supplementary File 1

Vignette 1

A 27 year old male presents with fever, sore throat and headache. Observations at triage show a pulse of 109 bpm, BP 127/88, RR 16, SpO2 98% and a temperature of 38.4°C. The patient has no significant PMH and denies regular medication except paracetamol. Examination reveals a well-nourished patient with no choryzal features. He has no neck stiffness or photophobia. There is erythema in the throat and mild exudate on both tonsils. His chest is clear to auscultation and abdomen soft and non-tender.

The four management options in this case were:

a) Redirect to own GP

b) Reassure, educate about viral illness, manage with hydration, rest and paracetamol. Follow up with own GP if not improving or worsening after 7-10 days.

c) Perform mono spot and FBC. Manage appropriately with results including antibiotics for tonsillitis, hydration and paracetamol. Follow up with own doctor in 7-10 days if worsening or not improving.

d) Start sepsis pathway and plan for admission.

Vignette 2

A 36 year old female presents to the Emergency Department with 48 hour left sided chest pain, left hand numbness and shortness of breath. Her father died of a myocardial infarction six months previously. She describes the pain as tightness made worse by deep breathing and left are movement. She denies any other past medical history or medication. Observations demonstrate a pulse of 101 bpm. ECG shows normal sinus tachycardia at 106bpm with no right axis deviation. Examination reveals and anxious, well-nourished female with a clear chest to auscultation. Here pain is reproducible on palpation, left arm movement and deep inspiration. She has no calf swelling or tenderness.

a) Redirect to own GP
b) Reassure and explore anxiety related symptoms and management. Discharge with advice and over the counter analgesia
c) Perform D-dimer to exclude pulmonary embolism and direct to ACU/CDU facility
d) Start ACS pathway including serial ECGs and cardiac markers and perform D-dimer to exclude pulmonary embolism. Direct patient to ACU/CDU facility

Vignette 3

A 22 year old female presents to the Emergency Department with a two day history of vulval rash and pain. She is sexually active with a new partner. She describes some hesitancy on initiation of micturition. Her observations are normal except for a slightly elevated temperature of 37.7°C. Examination reveals an erythematous uvula with multiple small painful ulcerated lesion.

a) Refer to own GP
b) Refer immediately to sexual health services for treatment
c) Treat with oral acyclovir or equivalent plus analgesia and refer to sexual health services
d) Perform FBC blood test, urine tests and refer to gynaecology

Vignette 4

A 33 year old male is referred to the Emergency Department by his GP for assessment of back pain with left leg numbness and difficulties with opening his bowels and passing urine with no incontinence. The patient is accompanied by his wife who is a consultant hepatologist. Both his wife and GP are concerned about the potential of cauda equine syndrome. The patient is a builder by trade and has had multiple episodes of back pain in the past. He has been taking co-codamol regularly for the last week.

The patients' observations are normal. On examination the abdomen is soft and non-tender with mild distension. His anal tone is normal with no saddle anaesthesia. The back is tender to palpation in the left lower lumbar area. Lower limb neurology demonstrates a reduction in hip flexion power (4/5) and a blunted left knee reflex and some paraesthesia over the lateral aspect of the lower leg.

a) Refer to own GP
b) Provide analgesia. Give advice about back strain, including rest, heat, analgesia and red flag features warranting re-attendance. Discharge home.
c) Perform bladder scan to exclude urinary retention. Ensure analgesia is effective +/- benzodiazepine. Discharge once pain is controlled and passing urine, with back pain advice. If pain remains uncontrolled admit for further management.
d) Perform urgent MRI, bladder scan +/-catheter and provide analgesia. Admit pending MRI results.
